# BTK modulates p73 activity to induce apoptosis independently of p53

**DOI:** 10.1038/s41420-018-0097-7

**Published:** 2018-09-11

**Authors:** Miran Rada, Nickolai Barlev, Salvador Macip

**Affiliations:** 1 Mechanisms of Cancer and Aging Laboratory, Department of Molecular and Cell Biology, University of Leicester, Leicester, UK; 2 Department of Biology, School of Science, Faculty of Science and Education Sciences, University of Sulaimani, Sulaimaniyah, Kurdistan Region, Iraq; 3 Institute of Cytology, RAS, Saint-Petersburg, Russia; 4 Present Address: Department of Surgery, McGill University Health Center Research Institute, Cancer Research Program, Montreal, Quebec Canada; 5 Moscow Institute of Physics and Technology, Dolgoprudny, Moscow region 117303 Russia

## Abstract

Bruton’s tyrosine kinase (BTK) is a key component of B cell receptor signalling. Because of this, BTK plays an important role in cell proliferation and survival in various B cell malignancies. However, in certain contexts, BTK can also have tumour suppressor functions. We have previously shown that BTK activates the p53 transcriptional activity by binding to and phosphorylating p53, as well as acting on MDM2 to reduce its inhibitory effects. This results in increased p53 functions, including enhanced cell death. Here, we report that BTK can also induce cell death and increase responses to DNA damage independently of p53. This is concomitant to the induction of p21, PUMA and MDM2, which are classic target genes of the p53 family of proteins. Our results show that these p53-independent effects of BTK are mediated through p73. Similar to what we observed in the p53 pathway, BTK can upregulate p73 after DNA damage and induce expression of its target genes, suggesting that BTK is a modulator of p73 functions and in the absence of p53. This effect allows BTK to have pro-apoptotic functions independently of its effects on the p53 pathway and thus play an important role in the DNA damage-related induction of apoptosis in the absence of p53. This provides a novel role of BTK in tumour suppression and contributes to the understanding of its complex pleiotropic functions

## Introduction

Bruton’s tyrosine kinase (BTK) is a cytoplasmic kinase that belongs to Tec family and is able to phosphorylate both serines and tyrosines^1^. BTK is a fundamental component of the B cell receptor signalling, through which it regulates many cellular processes, including differentiation and signalling^2^. Various types of leukaemia and lymphoma express abundant levels of active BTK^3,4^. Because of this, Ibrutinib, a small-molecule inhibitor of BTK that forms a covalent bond with BTK near the ATP binding site at cysteine 481 and blocks autophosphorylation^5^, has shown clinical efficiency against B cell malignancies^4^.

Despite its well-studied oncogenic role, it has been reported that BTK can also have tumour suppressor activity. For instance, we identified BTK in a screen of proteins selectively upregulated in senescence^6^. Also, BTK can induce cell death in several models^7–12^. The mechanisms by which BTK expression increases the percentage of cells undergoing apoptosis have not been completely elucidated. We showed that it could be mediated by its enhancing role on p53 activity, by binding to and phosphorylating both p53 and MDM2, and thus disrupting the negative feedback loop that leads to the proteasomal degradation of p53^12,13^. These novel roles of BTK suggest that it could contribute to the activation of other tumour suppressor pathways as well.

The p73 transcription factor belongs to the p53 family of proteins and has many splice variants, including transcriptional domain-containing p73 (TAp73) and amino deleted p73 (ΔNp73)^14,15^. TAp73 is a bona fide tumour suppressor; conversely, ΔNp73 shows oncogenic properties, inhibiting TAp73 and p53 functions^16^. TAp73 induces target genes that have a key role in cell cycle arrest and apoptosis, including p53 targets such as p21, Puma^17^, and MDM2^18,19^. MDM2 suppresses p73 activity, establishing a negative feedback loop similar to the one observed with p53^20^.

In order to better understand the tumour suppressor effects of BTK, we studied the mechanisms involved in the BTK-mediated induction of apoptosis. Here, we report that BTK can trigger cell death and enhance it after damage, independently of its effects on the p53 pathway. We show this is mediated by p73, the protein levels of are also regulated by BTK. We propose that BTK has a simultaneous impact on several pro-apoptotic pathways, among them p53 and p73, and that it is therefore an important component of tumour suppressor pathways.

## Results

### BTK causes and enhances cell death independently of p53

**Fig. 1:**
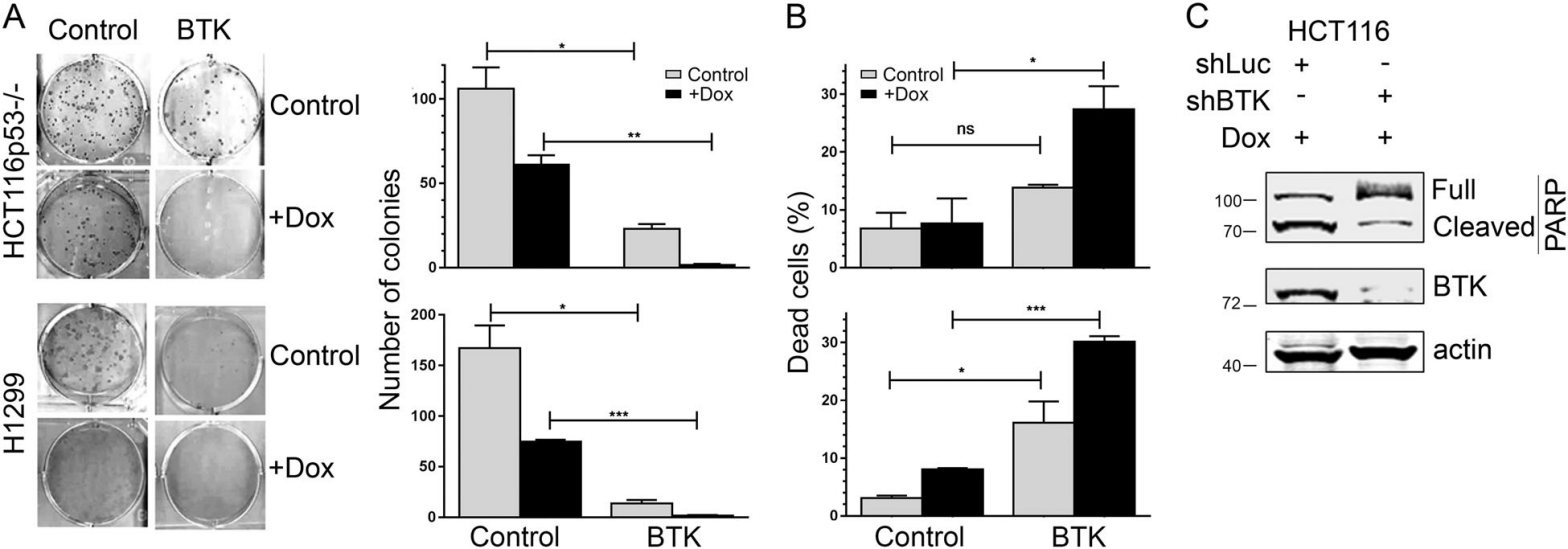
BTK induces apoptosis independently of p53. **a** Representative images of a colony formation assay of HCT116p53^−/−^ and H1299. A total of 100–200 cells were plated an incubated for 14 days before giemsa staining, in the presence or absence of 1.5 µM doxorubicin. Graphs show average and standard deviations of colony counts of three independent experiments performed in duplicates. *P* values for all experiments (*t*-student): ns: > 0.05; *: < 0.05; **: < 0.01; ***: < 0.001. **b** Percentage of dead cells, as measured by Annexin V staining, in HCT116p53^−/−^ and H1299 transfected with an empty vector (pCDNA3) or a BTK expression vector and treated or not with 1.5 µM doxorubicin for 48 h, 24 h after transfection. Graphs show average and standard deviations of three independent experiments. **c** Representative Western blot of lysates of HCT116p53^−/−^ stably transfected with an shRNA against luciferase (shLuci, control) or BTK (shBTK), showing levels of PARP and BTK. B-actin is used as a loading control

To study the p53-independent tumour suppressor functions of BTK, we expressed it in H1299, a p53 null non-small-cell lung carcinoma cell line, and a HCT116 colon cancer cell line with a p53 deletion (HCT116 p53^−/−^). As shown in Fig. 1a, BTK expression was able to significantly reduce to the formation of colonies in these cells and, when cells were exposed to DNA damaging agents, these effects were further increased. When propidium iodide (PI) was used to measure the presence of dead cells, we observed that BTK alone induced moderate levels of death (up to around 15%), but greatly increased the percentages after treatment with DNA damaging drugs (Fig. 1b). This indicates that the growth inhibition induced by BTK in the colony formation assay was caused by an increase in cell death. Consistent with this, Fig. 1c shows that BTK inhibition using shRNA reduced apoptosis induction by doxorubicin, as measured by PARP cleavage, suggesting that BTK is important for the responses to DNA damage both in the presence^12^ and in the absence of p53. These results together indicate that BTK has pro-apoptotic effects and can enhance cell death responses triggered by DNA damage, even when p53 is not present.

### BTK induces p21, PUMA and MDM2 expression independently of p53

**Fig. 2:**
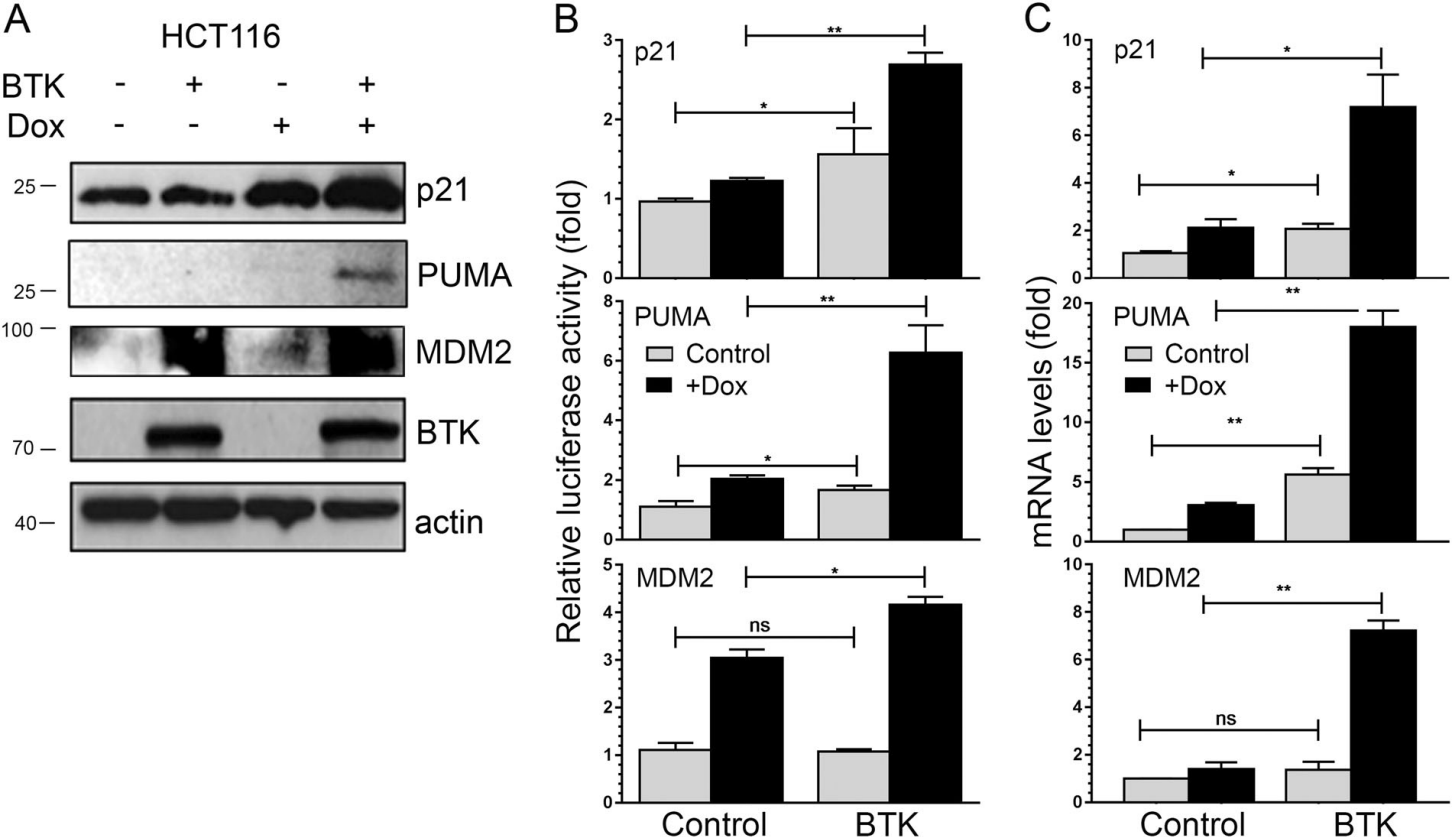
BTK increases the expression of p21, PUMA and MDM2 in the absence of p53. **a** Representative Western blot of lysates of HCT116p53^−/−^ transfected with BTK (+) or an empty vector (−) and treated or not with 1.5 µM doxorubicin for 48, 24 h after transfection, showing levels of p21, PUMA, MDM2 and BTK. B-actin is used as a loading control. **b** Relative luciferase activity of p21, PUMA and MDM2 reporters in HCT116p53^−/−^ transfected 24 h before with an empty vector (pCDNA3) or a BTK expression vector, in the absence or presence of 1.5 µM doxorubicin for 24 h. Graphs show average and standard deviations of three independent experiments. **c** mRNA levels, as measured by qRT-PCR, in the same cells. Graphs show average and standard deviations of three independent experiments

In order to better understand how BTK performs its functions, we next explored the induction of genes that we have previously seen upregulated by BTK through its effects on the p53 pathway, such as p21, PUMA and MDM2^12^. As shown in Fig. 2a, p21, PUMA and MDM2 protein levels were changed by BTK expression after DNA damage. Consistent with this, luciferase reporters showed that p21, PUMA and MDM2 all responded to BTK in the presence of DNA damage (Fig. 2b and Supplementary Figure 1A). This was also confirmed at the mRNA level (Fig. 2c). This shows that BTK increases the expression of these three genes. These data are consistent with the results shown in Fig. 1, and suggest that BTK acts on tumour suppressor pathways, especially after DNA damage, and that this is solely not dependent on its effects on p53.

### The p53-independent effects of BTK are mediated by p73

**Fig. 3:**
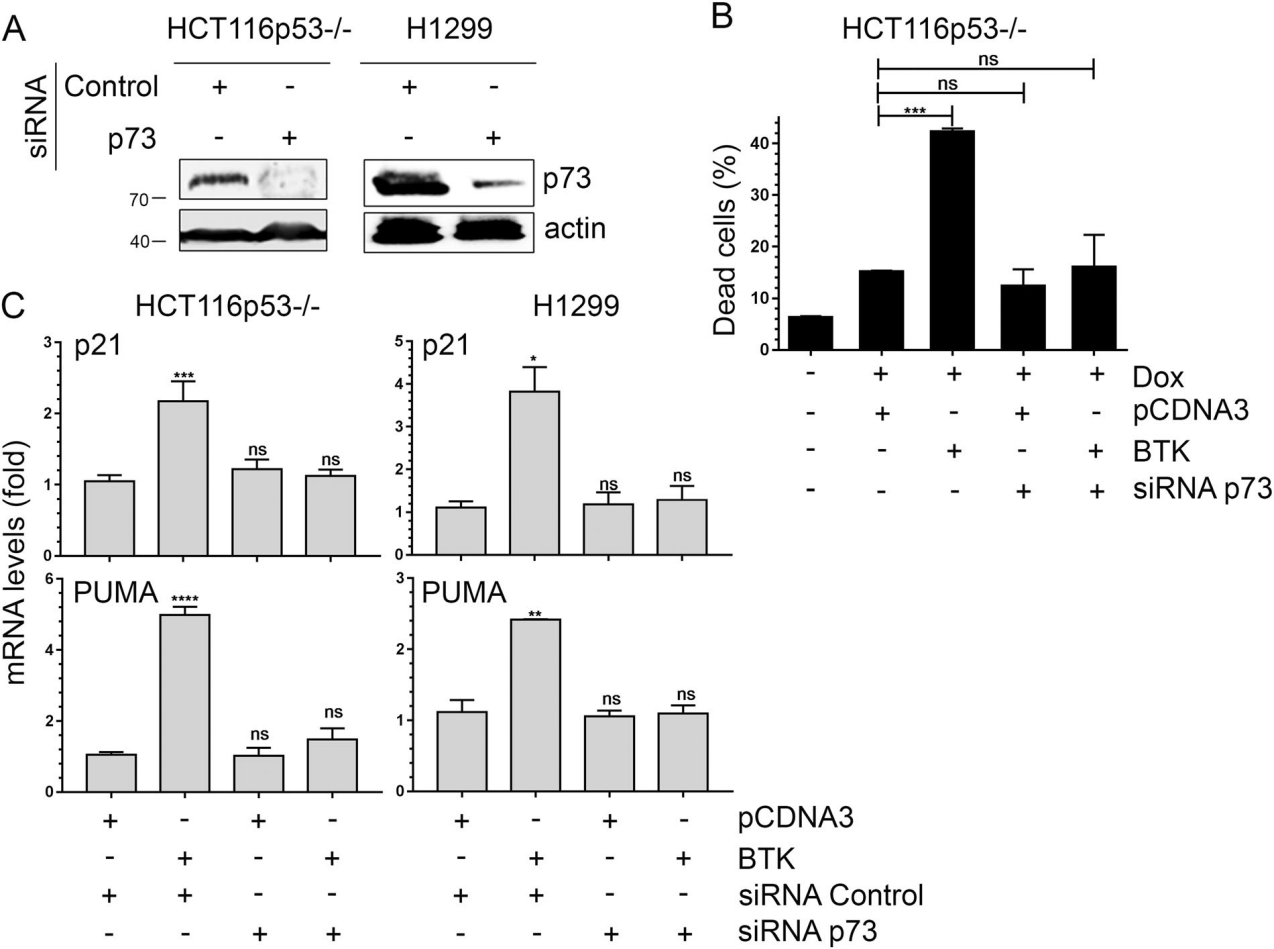
Inhibition of p53 blocks the p53-independent pro-apoptotic activity of BTK. **a** Representative Western blot of lysates of HCT116p53^−/−^ and H1299 transfected with control or p73 siRNA for 3 days, showing p73 protein levels. **b** Percentage of dead cells, as measured by Annexin V staining, in HCT116p53^−/−^ transfected with an empty vector (pCDNA3), a BTK expression vector and/or p73 siRNA, treated or not with doxorubicin, as described above. Graphs show average and standard deviations of three independent experiments. **c** mRNA expression of p21 and PUMA, as measured with qRT-PCR, in HCT116p53^−/−^ and H1299 transfected with an empty vector (pCDNA3), a BTK expression vector, p73 siRNA and/or a control siRNA. Graphs show average and standard deviations of three independent experiments performed in triplicate. Statistical significance is shown in comparison to the first column of each graph

Our data so far shows that, in p53 null cells, BTK has cellular effects that resemble those elicited by its modulation of the p53 pathway^12^. This provides a rationale to study the impact of BTK on other members of the p53 family, such as p73, that can also induce apoptosis and expression of similar target genes^21,22^. To test this hypothesis, we transfected HCT116p53^−/−^ and H1299 with siRNA against p73, which successfully inhibited the production of the protein (Fig. 3a). As shown in Fig. 3b, inhibition of p73 prevented the BTK-mediated enhancement of cell death after DNA damage. Consistent with this, BTK-mediated upregulation of p21 and PUMA was also blocked in the absence of p73 in p53 nulls cells (Fig. 3c). This suggests that, in the absence of p53, BTK depends on its effects on the p73 pathway to induce certain genes and the resulting apoptotic signals.

### BTK modulates p73 activity

**Fig. 4:**
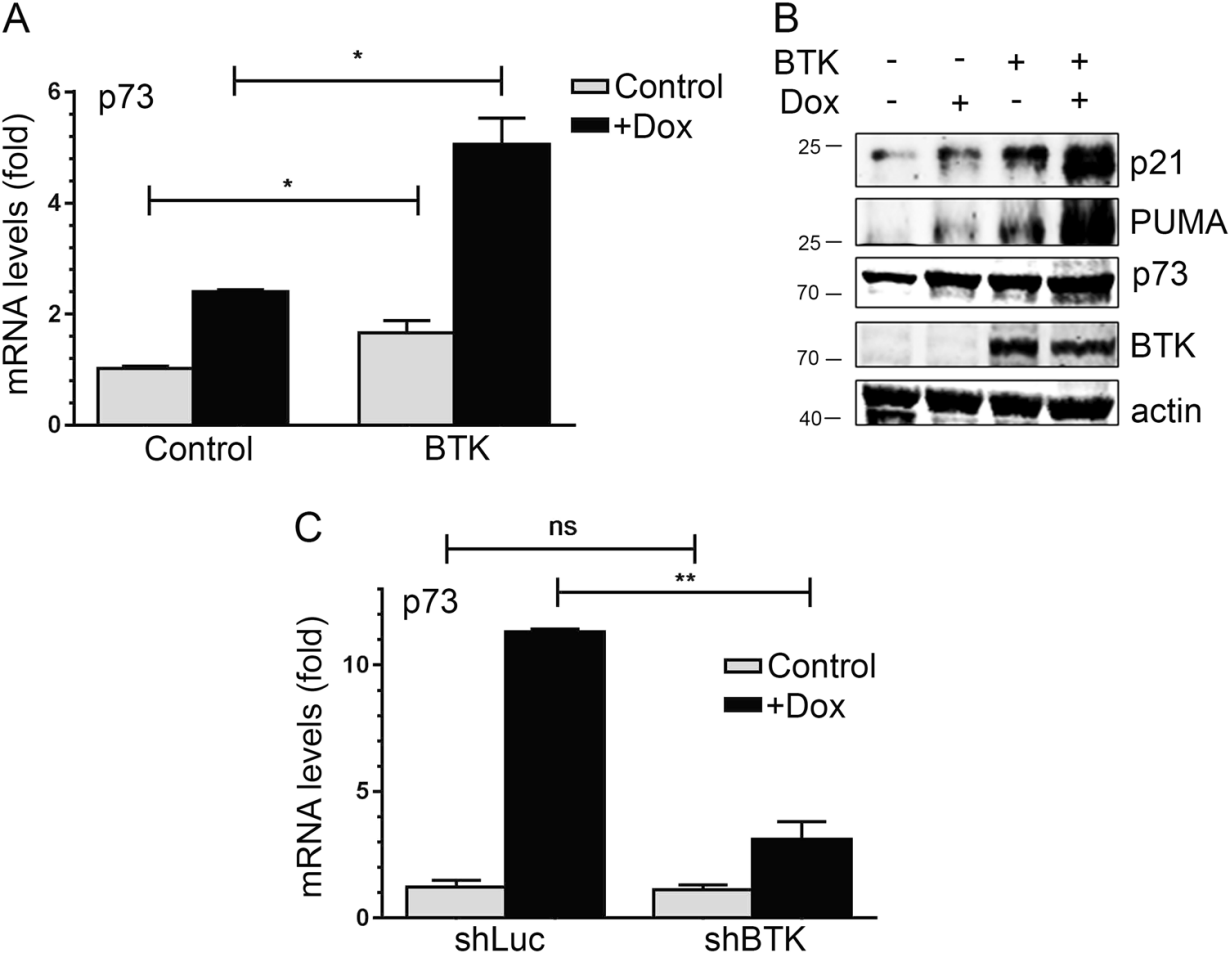
A positive feedback loop between BTK and p73. **a** mRNA expression of p73, as measured with qRT-PCR, in HCT116p53^−/−^ transfected with an empty vector (pCDNA3) or a BTK expression vector, in the absence or presence of 1.5 µM doxorubicin for 24 h. Graphs show average and standard deviations of three independent experiments performed in triplicate. **b** Representative Western blot of lysates of HCT116p53^−/−^ transfected with a empty vector (−) or a BTK expression vector (+) 24 h before being treated or not with 1.5 µM doxorubicin for 24 h, showing p21, PUMA, p73 and BTK protein levels. **c** mRNA expression of p73, as measured with qRT-PCR, in HCT116p53^−/−^ transfected with a control shRNA (shLuci) or an shRNA against BTK expression vector, in the absence or presence of 1.5 µM doxorubicin for 24 h. Graphs show average and standard deviations of three independent experiments performed in triplicate

We have described that BTK is an important regulatory factor in the p53 pathway, directly^12^ and through its interaction with MDM2^13^. Our results suggest that this may also be the case for p73. To test this hypothesis, we first studied whether p73 expression was regulated by BTK. We found that p73 increased after BTK upregulation both at the mRNA (Fig. 4a) and protein levels (Fig. 4b). This is consistent with BTK being able to inhibit the activity MDM2^13^, which is a negative regulator of p73^21^. Finally, we used an HCT116p53^−/−^ with a stable knockdown of BTK mediated by an shRNA. As shown in Fig. 4c, we observed that the absence of BTK significantly reduced the induction of p73 after DNA damage. This was accompanied by a reduction of p73 functions, as measured by the induction of target genes (Supplementary Figure 1B). This suggests that, in agreement with what we observed for p53, BTK is involved in the upstream regulation of p73, being important in its induction and activity after DNA damage.

## Discussion

The regulation of the cellular responses to damage involve different signalling pathways acting in coordination. The full extent of the elements involved in this complex network is not fully understood. Central to these stress responses is tumour suppressor p53 and its related family member, p73. The modulation of p53 and p73 activity is a topic of debate, and several models have been put forward to describe it^23^. However, new discoveries make it necessary to review such explanations routinely. We recently identified BTK as a novel member of the p53 pathway that has a strong impact on p53 activity. We showed that BTK, a kinase better known for its pro-oncogenic signals in leukaemia, is part of a positive feedback loop activated in stress responses that includes phosphorylation of both p53 and MDM2 to stabilise p53 protein levels^12,13^. These studies suggested that, outside BCR signalling, BTK could phosphorylate different members of the DNA damage networks.

In this report, we have further characterized the role of BTK in tumour suppressor pathways by studying its relationship with p73. p73 shares many functions with other members of its family of proteins, especially p53^22^, and can induce similar target genes. Our results suggests that BTK may be using p73 as a fail-safe mechanism to supplement or even substitute p53 functions if needed. BTK has a moderate pro-apoptotic activity on its own, which is still not fully understood, but it is more efficient at enhancing cell death signals from other effectors, such as p53 or p73. We found that, in the absence of p53, BTK can activate p73 and its target genes to induce cell death.

Several issues still need to be resolved to fully understand the relationship between BTK and p73. It is likely that BTK binds to and phosphorylates p73, similar to what happens with p53, but this would have to be confirmed and, if that is the case, the exact residues would have to be determined. Also, it would be important to determine whether BTK is a target gene of p73, like it is of p53. Understanding which particular stress situations activate a potential BTK-p73 loop, especially related to the p53 status of the cells, would be useful as well. Eventually, all these experiments should allow us to better understand how p53 and p73 are regulated and which common features they share, in order to describe a more complete picture of how the tumour suppressor mechanisms protect us against the emergence of transformed cells.

## Materials and methods

### Cell culture

Cells were cultured in DMEM and incubated at 37 °C with 5% CO_2_. Media was changed every 24–48 h, depending on cell confluency. Transfection was carried as previously described^13^. Furthermore, 1.5 μM doxorubicin (Sigma-Aldrich) was added for 24 h to induce DNA damage.

### Plasmids constructs

pGL3-p21, pGL3-PUMA, pGL3-MDM2 (a gift from Dr David W Meek, University of Dundee, UK), pSV-β-Galactosidase, pCDNA3, pCDNA3-FLAG-MDM2, pCDNA3-GFP-BTK (OriGene, #RG211582), pGEX-MDM2, pGEX-UBC9, shBTK (Santa Cruz sc-29841-sh), and shRNA against luciferase (Sigma Aldrich SHC007, Mission pLKO.1puro Luciferase) were used in this study.

### BTK silencing

To generate cells with a stable downregulation of BTK, a shRNA against BTK was used. A shRNA against luciferase was used as a control. The shRNA was transfected into HCT116p53^−/−^ using Turbofect, following manufacturer’s protocols. The medium in the plates was changed after an overnight incubation and 2 μg/ml puromycin added to each plate to select for transfected cells. Cells were kept under selection for 2 weeks.

### p73 silencing

Small interfering RNA (siRNA) against p73 was used to transiently downregulate p73 expression. HCT116p53^−/−^ were transfected with siRNA (Santa Cruz, #sc-36167) using Lipofectamine 2000, following manufacturer’s protocols. A scrambled siRNA was used as control.

### Luciferase reporter assay

A total of 100,000 cells per well were seeded in 24-well plates one day before transfection. Furthermore, 1.0 μg of total plasmid DNA per well was used, 24 h after transfection, media was replaced by fresh growth media and samples was treated with 1.0 μM Doxorubicin. Furthermore, 20 h later, lysates were collected in lysis buffer and stored at −80 °C overnight. One quarter of the 80 μl cell lysate was then analysed with a luciferase assay kit (BioVision, UK) using Victor X3 Multilabel Plate Reader (Perkin Elmer, UK) and following manufacturer’s instructions.

### Immunoblot analysis

Lysates were extracted as previously described^13^. Primary antibodies used: β-actin (Abcam, #ab8227), p73 (Santa Cruz, #sc-7957), p21 (Santa Cruz Biotechnology, #sc-53870), PUMA (Cell Signalling, #4976), BTK (Cell Signalling, #8547 S), MDM2 (Abcam, #ab137413), and PARP (Cell Signalling, #9542 S).

### Quantitative real time PCR (qRT-PCR)

Expression of MDM2, p21, p73 and Puma genes was analysed by RT-PCR as previously described^24^. The following primers were used: (MDM2) GTTCTTTTTTATCTTGCCCAGTATATT (FWD), GTGCTCTTTCACAGAGAAGCTTG (REV); (p21) CACCGAGACACCACTGGAGG (FWD), GAGAAGATCAGCCGGCGTTT (REV), (p73) AGCAGCCCATCAAGGAGGAGTT (FWD), TCCTGAGGCAGTTTTGGACACA (REV); (PUMA) GACCTCAACGCACAGTACGA (FWD), CACCTAATTGGGCTCCATCT (REV); (GAPDH) GGGAAGGTGAAGGTCGGAGT (FWD), TTGAGGTCAATGAAGGGGTCA (REV).

### Cell death

Cell death was measured using PI as previously described^13^. Flow cytometry analysis was performed using Becton Dickinson FACSanto II FACSDiva 6.0 software (Becton Dickinson)

### Colony formation assay

The colony formation assay was performed as previously described^12^.

## Electronic supplementary material

Supplementary Figures

Author contribution form
