# Supplementary material for: BTK modulates p73 activity to induce apoptosis independently of p53
Source: Cell Death Discov. 2018 Sep 11;4:95. doi: 10.1038/s41420-018-0097-7 (PMC6134113; doi:10.1038/s41420-018-0097-7)
Supplement: Supplementary file 1 — Supplementary Figures [file 41420_2018_97_MOESM1_ESM.pdf]

SUPPLEMENTARY FIGURES

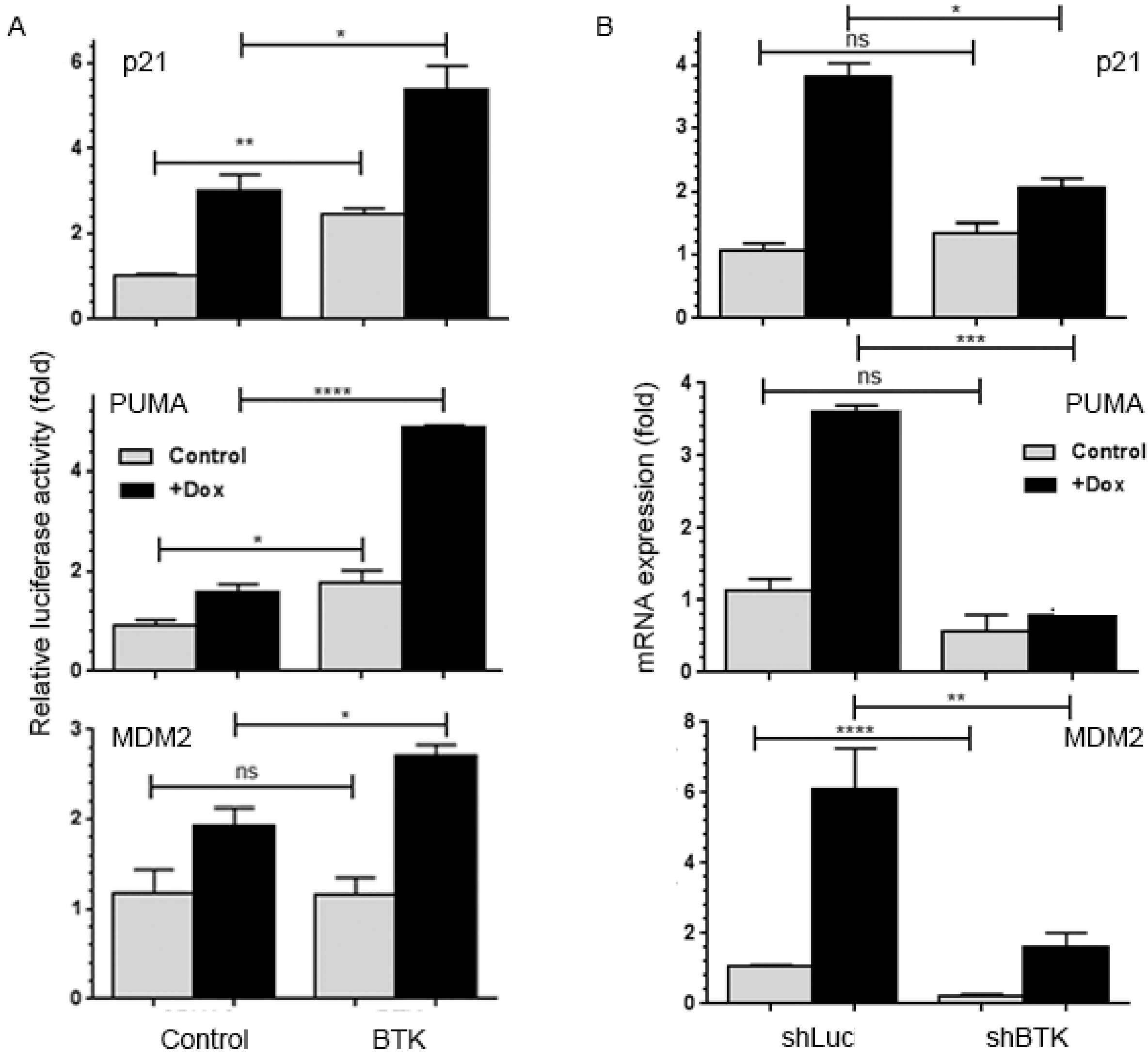

Figure S1. (A) Relative luciferase activity of p21, PUMA and MDM2 reporters in H1299 transfected 24h before with an empty vector (pCDNA3) or a BTK expression vector, in the absence or presence of 1.5µM doxorubicin for 24 hours. Graphs show average and standard deviations of three independent experiments. **(B)** mRNA levels of p21, PUMA and MDM2, as measured by qRT-PCR, in HCT116p53$^{-/-}$ from the experiment in Figure 4C Graphs show average and standard deviations of three independent experiments.
