# Supplementary material for: BTK modulates p73 activity to induce apoptosis independently of p53
Source: Cell Death Discov. 2018 Sep 11;4:95. doi: 10.1038/s41420-018-0097-7 (PMC6134113; doi:10.1038/s41420-018-0097-7)
Supplement: Supplementary file 2 — Author contribution form [file 41420_2018_97_MOESM2_ESM.pdf]

DECLARATION OF CONTRIBUTIONS TO ARTICLE

ADMC

Manuscript Number:	Journal Name:	
CDDIS-18-1949	*Cell Death & Disease*	(the 'Journal')

Proposed Title of the Contribution:

BTK modulates p73 activity to induce apoptosis independently of p53 (the 'Contribution)

Author(s):

Miran Rada, Nickolai Barlev, Salvador Macip (the 'Authors')

For all *CDDis* articles, each person named as an author in the published version must be able to show he or she has contributed substantially to the article.

Authorship credit should be based on 1) substantial contributions to conception and design, acquisition of data, or analysis and interpretation of data; 2) drafting the article or revising it critically for important intellectual content; and 3) final approval of the version to be published. Authors should meet conditions 1, 2 and 3.

Any person who cannot be shown to have made a substantial contribution to the article cannot be listed as an author in the final version. The name of any person who is deemed to have made a minor contribution can, however, appear in the Acknowledgments section of the article.

Please complete the table below to indicate the contributions of all named authors to the manuscript.

Author Full Name:	Specification of Contribution to the Manuscript:
Miran Rada	Designed the experiments, analysed the data and prepared the manuscript. Performed the experiments.
Nickolai Barlev	Designed the experiments, analysed the data and prepared the manuscript.
Salvador Macip	Designed the experiments, analysed the data and prepared the manuscript.

Please complete the table below to indicate the contributions of all named authors to the figures.

Figure 1:

All experiments performed by MR.
Data analysed by MR, NB and SM.
Figure prepared by MR and SM.

Figure 2:

All experiments performed by MR.
Data analysed by MR, NB and SM.
Figure prepared by MR and SM.

Figure 3:

All experiments performed by MR.
Data analysed by MR, NB and SM.
Figure prepared by MR and SM.

Figure 4:

All experiments performed by MR.
Data analysed by MR, NB and SM.
Figure prepared by MR and SM.

Figure 5:

Figure 6:

Signed for and on behalf of the Author(s):	Print Name:	Date:
	Salvador Macip	02/07/18
